# Current Perspectives on Tooth Implantation, Attachment, and Replacement in Amniota

**DOI:** 10.3389/fphys.2018.01630

**Published:** 2018-11-21

**Authors:** Thomas J. C. Bertin, Béatrice Thivichon-Prince, Aaron R. H. LeBlanc, Michael W. Caldwell, Laurent Viriot

**Affiliations:** ^1^Team Evolution of Vertebrate Dentition, Institut de Génomique Fonctionnelle de Lyon, Université de Lyon, CNRS UMR 5242, Ecole Normale Supérieure de Lyon, Université Claude Bernard Lyon 1, Lyon, France; ^2^Faculte d’Odontologie, Université Claude Bernard Lyon 1, Lyon, France; ^3^Service d’Odontologie, Hospices Civils de Lyon, Lyon, France; ^4^Department of Biological Sciences, University of Alberta, Edmonton, AB, Canada

**Keywords:** tooth implantation, tooth replacement, periodontium, amniota, evolution, thecodonty, pleurodonty, acrodonty

## Abstract

Teeth and dentitions contain many morphological characters which give them a particularly important weight in comparative anatomy, systematics, physiology and ecology. As teeth are organs that contain the hardest mineralized tissues vertebrates can produce, their fossil remains are abundant and the study of their anatomy in fossil specimens is of major importance in evolutionary biology. Comparative anatomy has long favored studies of dental characters rather than features associated with tooth attachment and implantation. Here we review a large part of the historical and modern work on the attachment, implantation and replacement of teeth in Amniota. We propose synthetic definitions or redefinitions of most commonly used terms, some of which have led to confusion and conflation of terminology. In particular, there has long been much conflation between dental implantation that strictly concerns the geometrical aspects of the tooth-bone interface, and the nature of the dental attachment, which mostly concerns the histological features occurring at this interface. A second aim of this work was to evaluate the diversity of tooth attachment, implantation and replacement in extant and extinct amniotes in order to derive hypothetical evolutionary trends in these different dental traits over time. Continuous dental replacement prevails within amniotes, replacement being drastically modified only in Mammalia and when dental implantation is acrodont. By comparison, dental implantation frequently and rapidly changes at various taxonomic scales and is often homoplastic. This contrasts with the conservatism in the identity of the tooth attachment tissues (cementum, periodontal ligament, and alveolar bone), which were already present in the earliest known amniotes. Because the study of dental attachment requires invasive histological investigations, this trait is least documented and therefore its evolutionary history is currently poorly understood. Finally, it is essential to go on collecting data from all groups of amniotes in order to better understand and consequently better define dental characters.

## Introduction

Owning true teeth is one of the great innovations of vertebrates. The acquisition of a dentition has led to a diversification of predation and oral processing patterns that can, at least in part, explain the tremendous evolutionary success of vertebrates (Pough et al., 2009). Today, vertebrates display a great diversity of dentitions in terms of tooth location, number, shape, occlusion, attachment, implantation, and replacement (Ungar, 2010; Berkovitz and Shellis, 2017). Teeth are highly mineralized organs that consist of the hardest known tissues, dentin and especially enamel, composed of, respectively, 70 and 96% mineral matrix (Nanci, 2003). As a result, teeth are of great interest in zoology and especially in paleontology for two reasons: (1) many fossils are known only through their teeth because dental remains are more resistant to decay than the rest of the skeleton; and (2) teeth often possess diagnostic features that allow for identification of a fossil to generic or even specific levels.

Features such as tooth replacement and tooth implantation and attachment are particularly studied in Amniota because of their importance in the systematics and evolutionary relationships among various clades. Tooth replacement corresponds to the succession of a tooth by another tooth at a given position. By extension, continuous dental replacement consists of a series of dental generations that sequentially occur at the same location over the life of the animal, which defines a tooth family. Tooth implantation concerns the geometrical organization of the interface between the tooth and the tooth-bearing element. Teeth could be implanted either in a shallow (subtheconty) or deep (thecodonty) alveolus, or on the lingual side of the labial wall of the jaw bone (pleurodonty), or on the margin of the jawbone (acrodonty) (Owen, 1845; Romer, 1956; Edmund, 1969). The nature of tooth attachment to the tooth-bearing element also is a meaningful morphological feature to consider in the frame of odontological studies as a tooth could either be firmly anchored to the tooth bearing element (ankylosis) or linked to it through a soft periodontal ligament (gomphosis).

While this terminology has been widely used to describe the tooth-bone interface in both extant and extinct species, the historical definitions and the usage of the various terms raise several problems. First, the current terminology defines specific types of attachment and implantation of teeth, but does not encompass the biological diversity of ways in which teeth are anchored to the jaws in amniotes. As a result, each discovery of new geometries of dental implantation or new modes of attachment that do not fit seamlessly into the predefined categories often leads to the creation of new subcategories (Smith, 1958; Sues and Olsen, 1993). Second, definitions of categories or subcategories may vary considerably according to the authors. Third, certain types of dental implantation include in their definitions various other characteristics, some of which are related to dental attachment. These considerations mix geometrical and histological features, which may cause confusion between two characteristics that are not completely interdependent. All these problems complicate a study of tooth attachment and implantation, as the comparison between different authors may be misleading.

Here we review the historical and modern literature on tooth replacement, implantation, and attachment in Amniota, with the primary goal of precisely defining or redefining the associated terminology. We also aimed to illustrate many ambiguous cases so that the diversity of dental replacement, implantation, and attachment are taken into account. We used a comprehensive approach broken down into six major morphological features: (1) the implantation geometry, (2) the histological nature of the attachment, (3) the replacement mode, (4) the number of tooth generations, (5) the path of replacement teeth, and (6) the resulting resorption patterns. As these six features display significant variation among extant and extinct Amniota, we will first present an overview of this diversity in extant amniotes in order to demonstrate the interrelationships between these six major characteristics and their modifications over the course of evolution.

## Composition of the Periodontium in Amniota

Historically, the tissues that are responsible for tooth attachment to the jaws in amniotes have been termed the cementum, the periodontal ligament, the alveolar bone, the “attachment bone,” and the interdental bone.

### Cementum

**FIGURE 1:**
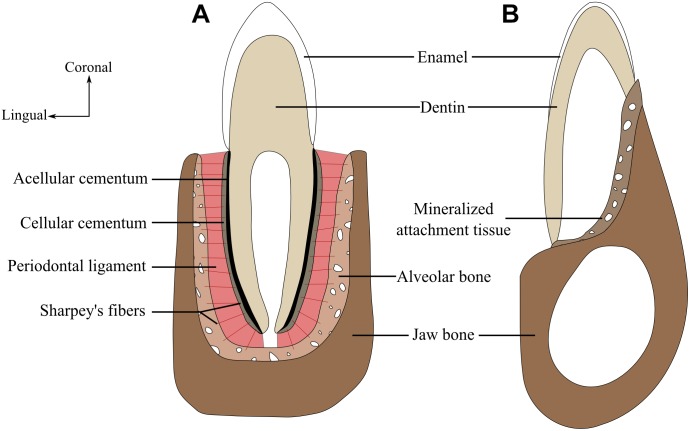
The amniote periodontium and tooth tissue organization. **(A)** Labio-lingual section of a tooth with a thecodont implantation and gomphosis attachment. **(B)** Labio-lingual section of a tooth with a pleurodont implantation and ankylosed attachment. Modified from (LeBlanc and Reisz, 2013).

The cementumis a mineralized tissue layer that covers the base of the tooth (Figure 1). Its composition is very similar to that of bone, being approximately 50% hydroxyapatite and 50% collagen (mainly type I collagen) and non-collagenous proteins (Saygin et al., 2000). However, and contrary to the bone, cementum exhibits little to no remodeling, no nerves, and usually no vascular system. Cementum is produced by cementoblasts differentiated from dental follicles ofectomesenchymal origin, or from the Hertwig epithelial root sheath (HERS) (Diekwisch, 2001; Yagyuu et al., 2010). Cementum can be very thick and form a root-like structure, as documented in mosasaurid squamates (Caldwell et al., 2003; Luan et al., 2009; LeBlanc et al., 2017b) and ichthyosaurs (Maxwell et al., 2011b). Cementum can be cellular or acellular (Figure 1). Acellular cementum contains no cell bodies whereas cellular cementum contains cementocytes that, similar to osteocytes in bone, are simply cementoblasts entombed in the cementum matrix they produced. Cellular and acellular cementum often are neighboring tissues (Figure 1); cellular cementum overlies acellular cementum, which in turn directly coats the dentin of the roots (Budney et al., 2006). Finally, cementum may be afibrillar, or include either extrinsic fibers that are perpendicular to its outer surface and in continuity with periodontal ligament fibers, or intrinsic fibers that are parallel to its outer surface (Saygin et al., 2000; Grzesik and Narayanan, 2002).

### Periodontal Ligament

In some amniotes, the periodontal ligament acts as a link between cementum and alveolar bone (Figure 1). The periodontal ligament is a soft tissue composed of collagen fibers produced by fibroblasts. Development of the periodontal ligament is associated with a perforation of the HERS (McIntosh et al., 2002). Some of the collagen fibers are partially embedded in the alveolar bone on one side and into the cementum on the other side, forming partially mineralized Sharpey’s fibers. The periodontal ligament itself, however, can be partially or fully mineralized (McIntosh et al., 2002). The former presence of a soft ligament is difficult to evaluate in fossil specimens because a ligament does not fossilize. Decay of the ligament during fossilization, however, creates a periodontal space around the tooth roots, which may indicate the former presence of a ligament. The periodontal space can lead to post-mortem tooth shedding, but it can also be infiltrated during diagenesis by sediment or mineral-rich fluids that will crystallize and preserve the tooth root *in situ* (LeBlanc et al., 2016a). Another line of evidence of the former presence of a periodontal ligament is the occurrence of Sharpey’s fibers either into alveolar bone or into root cementum, or both (Jones and Boyde, 1974). The absence of a periodontal space is marked by a fusion between cementum and alveolar bone, making them nearly indistinguishable (Caldwell et al., 2003; Luan et al., 2009; LeBlanc and Reisz, 2013).

### Alveolar Bone

Among extant amniotes, alveolar bone has been almost exclusively investigated in Mammalia and Crocodilia (McIntosh et al., 2002), the most thorough studies being conducted in mice and humans. Alveolar bone (Figure 1) is a specialized part of the tooth-bearing element that forms the primary support structure of teeth (Saffar et al., 1997; Sodek and Mckee, 2000). Although it has a specialized function, the basic cellular and matrix components of alveolar bone are consistent with other bone tissues (Sodek and Mckee, 2000). When compared to the rest of the tooth-bearing elements, alveolar bone has a different origin because it derives from layers of ecto-mesenchymal cells surrounding the base of the dental papilla (Ten Cate et al., 1971). As alveolar bone develops concomitantly with dental development and eruption (Kjaer and Bagheri, 1999; Matalová et al., 2015), a proper delimitation of alveolar bone can only be achieved through detailed observations of dental development (Ten Cate et al., 1971; Tencate and Mills, 1972). Here we consider the alveolar bone as the bone volume that is resorbed and redeposited through dental replacement cycles (following LeBlanc and Reisz, 2013, 2015). The primary structure of the alveolar bone is woven-fibered in both mammals and crocodilians (Caldwell et al., 2003; Budney et al., 2006). The alveolar bone remains woven fibered throughout life in crocodilians, whereas it is remodeled to lamellar bone in mammals when tooth replacement stops (Yeh and Popowics, 2011; LeBlanc and Reisz, 2013). Alveolar bone provides the attachment site for Sharpey fibers. These collagen fibers are organized into bundles and calcified fibers within the bone in order to provide a strong tooth-bone attachment. This portion of alveolar bone is sometimes referred to as bundle bone due to the presence of these fiber bundles (Chu et al., 2014).

### Attachment Bone

**FIGURE 2:**
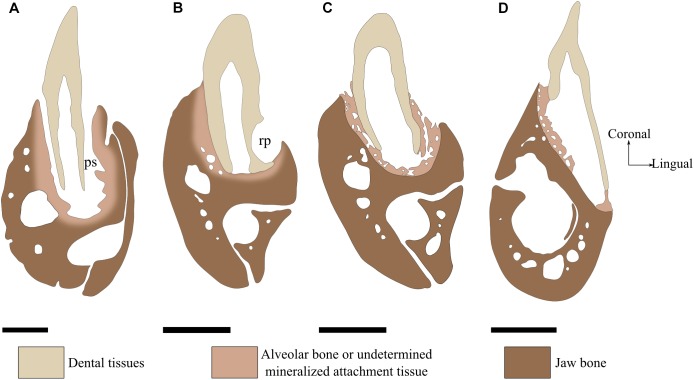
Tooth attachment illustrated by labio-lingual sections of mandibles in various species. **(A)** Gomphosis attachment associated with a thecodont implantation in *Crocodylus niloticus*. Note that the tooth and the bone are not in direct contact, leaving a periodontal space (ps). As the limit between alveolar bone and jaw bone is difficult to define, it is drawn as blurred. **(B)** Ankylosis attachment associated with a subthecodont implantation in *Tupinambis teguxin* (specimen MNHN 1967-96). The tooth is fused to a mineralized attachment tissue, leaving no periodontal space. Note the presence of a replacement pit (rp). **(C)** Tooth newly attached in *Tupinambis teguxin* (same specimen). The “attachment bone” is mineralized, which makes it more clearly distinguishable from the dentary bone. **(D)** Ankylosis attachment associated with a pleurodont implantation in *Cyclura cornuta* (specimen MNHN 1919-45). Scale bar is 2 mm.

In most species presenting no soft ligament, the term “attachment bone” is used to describe the bone linking the tooth to the tooth-bearing element (Zaher and Rieppel, 1999; Rieppel, 2001). The attachment bone progressively mineralizes over dental replacement, being less mineralized than the tooth-bearing element at early stages (Figure 2C) and nearly indistinguishable from the tooth-bearing element at latter stages (Figure 2B). Similarly to the alveolar bone, the attachment bone is completely or partially resorbed and reformed throughout each dental replacement (Peyer, 1968; Zaher and Rieppel, 1999). For some authors, the fact that alveolar bone and attachment bone have similar functions make them homologous (Maxwell et al., 2011a). For other authors, attachment bone may be homologous with cementum (Edmund, 1969; Rieppel and Kearney, 2005; Luan et al., 2009). Here we will not consider attachment bone as a distinct tissue type because it is only defined after the role it plays in certain groups and not after its histological features.

### Interdental Bone

Interdental bone includes projections from the jawbone that separate teeth from each other (Zaher and Rieppel, 1999). Some authors used the structure of the interdental bone to define tooth implantation (Luan et al., 2009; Dumont et al., 2016). Other authors suggested that interdental bone is not a distinct tissue, but consists of remains of various dental tissues (Caldwell et al., 2003; Budney et al., 2006; LeBlanc et al., 2017a,b). Detailed histological investigations of the interdental bone have revealed a structural heterogeneity linked to the persistence of dental tissues over multiple replacement cycles and are linked to tooth migration through ontogeny (LeBlanc et al., 2017a). Here we will neither consider the interdental bone as a distinct bone structure nor regard its structure as relevant to define tooth implantation.

## Terminology

### Morphology and Histology of the Tooth-Bone Interface

Criteria for determining implantation and attachment types are, respectively, based on morphological and histological characteristics of the tooth-bone interface.

#### Geometry of Implantation

Tooth implantation describes the various morphologies of the interface between the functional tooth (i.e., fully developed and implanted) and the bone, irrespective of the attachment.

##### Acrodonty

**FIGURE 3:**
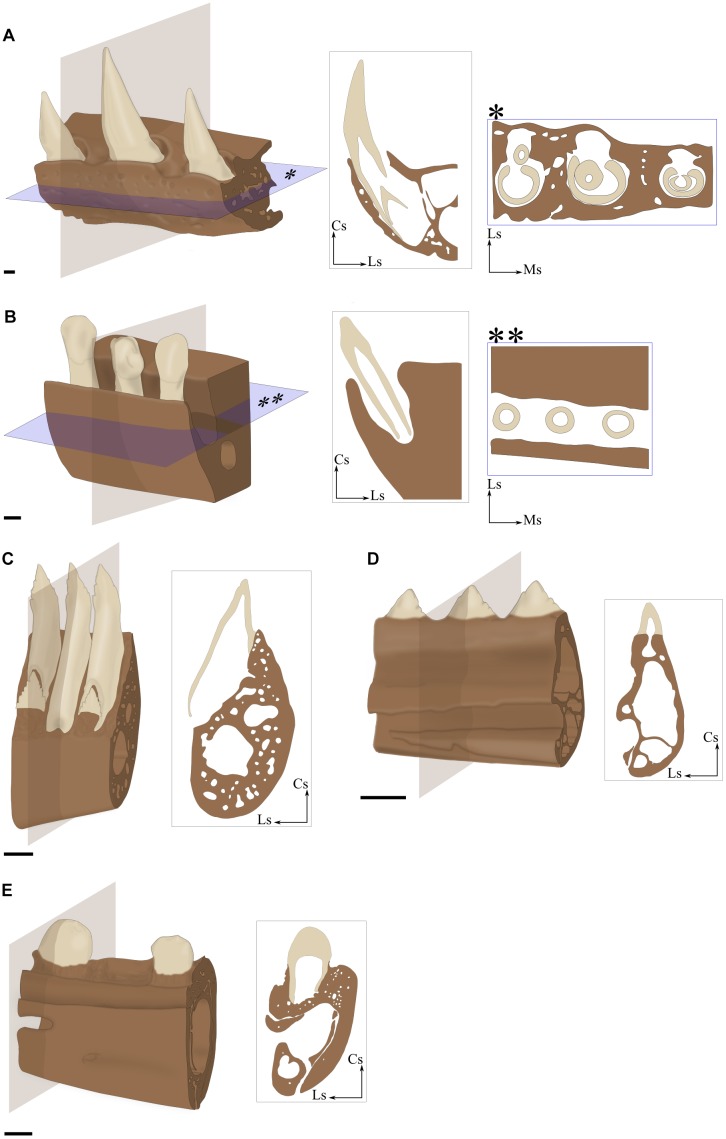
Implantation geometry illustrated by 3D portions of maxilla **(A,B)** and mandible **(C–E)** associated with virtual sections. **(A)** Thecodont implantation in the Nile crocodile (*Crocodylus niloticus*). **(B)** Aulacodont implantation in the Porpoise (*Phocoena* sp., specimen ENSL agSVSTUA 024181). **(C)** Pleurodont implantation in the Green iguana (*Iguana iguana*, specimen MNHN 1939-523). **(D)** Acrodont implantation in the Graceful chameleon (*Chamaeleo gracilis*, specimen MNHN 1942-114). **(E)** Subthecodont implantation in the Tegu (*Tupinambis teguxin*, specimen MNHN 1967-96). Cs, coronal side; Ls, lingual side; Ms, mesial side. Scale bar is 1 mm.

The apex of the tooth is set at the top (the Greek prefix *acro-* means at the extremity) of the tooth-bearing element, without any mediolateral tooth-bone contact (Figure 3D). The tooth is neither set in a groove nor in alveoli because no bony wall is present on any side of the tooth.

##### Aulacodonty

The tooth is set in a groove (*aulakos* in Greek) and its depth is at least equal to the height of the crown (Figure 3B). No bone separates adjacent tooth positions along the tooth-bearing element. The lingual and the labial (=vestibular) walls are roughly the same height. When Mazin (1983) defined this term to describe tooth implantation in Ichthyosauria, he assumed that aulacodonty was derived from thecodonty, but current knowledge of dental evolution in reptiles does not provide enough evidence to support this conclusion (Motani, 1997). Here, we follow Motani (1997) who recommends that this term be used for descriptive purposes, without evolutionary connotation.

##### Pleurodonty

The labial surface of the tooth is set against the labial side (the Greek prefix *pleuro-* means to the side) of the tooth-bearing element (Figure 3C). Whereas this is the main point of contact between the tooth and the jaw, other tooth-bone contact zones may exist around the tooth. According to Lessmann (1952), ‘labial pleurodonty’ involves the development of a basal bony lingual plate that supports lingual bases of teeth along the jaw (Figure 2D). This lingual bony plate may form a very shallow groove, which differs from aulacodont geometry (see above) by a clear difference in height between the labial and lingual walls. Dong (1972) and Presch (1974) considered that the presence of well-developed attachment bone surrounding the tooth base should be considered as a different type of implantation called subpleurodonty. Like other types of implantation, the pleurodont type must be based on a few unambiguous characters and the multiplication of subcategories in each particular case introduces more confusion than clarification. As a consequence, we will not distinguish any subcategory of pleurodonty in this review.

##### Subthecodonty

The use of the term ‘subthecodonty’ as well as differences between subthecodonty and thecodonty have been the subject of intense discussion (Motani, 1997; Zaher and Rieppel, 1999). Zaher and Rieppel (1999) notably protested against the retention of the term ‘subthecodonty’ and alternatively suggested instead to use ‘ankylosed thecodonty,’ arguing that thecodonty necessarily implies a ligament attachment. As we treat here implantation geometry of the tooth independently of its attachment, it does not seem inappropriate to us, as the latin term *theca* refers only to the socket in which the tooth sits. We here refer to subthecodont implantation in a geometrical sense, implying that the tooth is set in an asymmetrical and shallow socket (Figure 3E). Asymmetry is created by differences in height between the lingual and labial walls of jaw bones, the labial wall being higher than the lingual wall (Romer, 1956; Poole, 1967; Motani, 1997). Mesial and distal walls are also usually lower than the lingual wall. In some cases, the presence of alveolar bone makes the alveolus look symmetrical.

##### Thecodonty

This form of implantation has historically been linked to the teeth of mammals and crocodilians. As such, it has often been conflated with the ligamentous tooth attachment mode in these two groups. In our view, thecodonty occurs when the tooth is set in a deep and symmetrical alveolus (Figure 3A). The depth of the alveolus is at least equal to the height of the crown. The four bony walls of the alveolus have comparable heights, although minor differences may exist.

#### Nature of the Attachment

Teeth are attached to bone according to various types of connections between the tooth and the tooth-bearing element. We follow the interpretations of Caldwell et al. (2003) and LeBlanc et al. (2017a) that tooth attachment should be described independently from the tooth implantation geometry.

##### Gomphosis

The term originates from the Latin *gomphus*, meaning peg, and refers to the immovable joint between tooth and bone. The tooth is attached to the bone through a non-mineralized ligament that links the cementum to the alveolar bone (Figure 2A). Terminal ends of the ligament are mineralized (Sharpey’s fibers) and are inserted into the cementum and into the alveolar bone that forms the tooth socket. The presence of Sharpey’s fibers is, however, not a proof of gomphosis because the ligament may be completely mineralized over dental ontogeny (McIntosh et al., 2002; LeBlanc et al., 2016a). The only evidence of gomphosis in fossil specimens is the presence of a periodontal space resulting from the decay of the non-mineralized ligament. The periodontal space, however, can be secondarily infilled during diagenesis by sediment and mineral inclusions (LeBlanc et al., 2016a). The ligament can be more or less mineralized (McIntosh et al., 2002), but must retain a non-mineralized component between the cementum and the alveolar bone for it to be considered a gomphosis.

##### Ankylosis

This term is derived from the Greek *ankulos* that means constricted and refers to the decrease in the freedom of movement of a joint. Here the tooth is fused to the tooth-bearing element through mineralized tissues (Figures 2B–D). The fusion occurs between dental mineralized tissues (cementum or dentin) and alveolar bone (referred to as “attachment bone” by some) (Luan et al., 2009; Buchtová et al., 2013). Sometimes, ankylosis can be achieved through a mineralized periodontal ligament (Caldwell et al., 2003; Luan et al., 2009; LeBlanc et al., 2016a, 2017b).

### Replacement Types

As stated by Luo et al. (2004) concerning the study of cynodont-mammal evolution, patterns of dental replacement can be broken down to several basic morphological elements that are (1) the number of successional dental generations at each tooth locus, (2) the direction of replacement, and (3) the mode of replacement. For the present review, we retained these points and we added the resulting resorption pattern.

#### Number of Tooth Generations

##### Polyphyodonty

**FIGURE 4:**
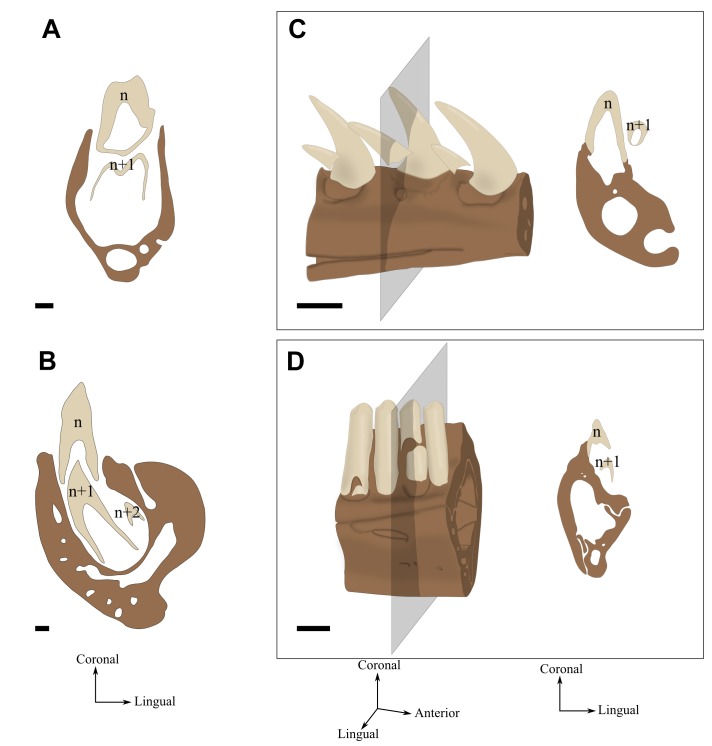
Tooth replacement illustrated by labio-lingual sections and perspective views of mandibles in various species. *n*, *n*+1, and *n*+2 indicate which generation teeth belong. **(A)** Diphyodonty in the rabbit (*Oryctolagus cunniculus*). **(B)** Polyphyodonty in the Nile crocodile (*Crocodylus niloticus*). **(C)** Labio-mesial replacement in *Zamenis* sp. (specimen MNHN 1969-789). **(D)** Labio-vertical replacement in *Lacerta viridis* (MNHN 1887-813). Coronal sections correspond to the gray section plan. Scale bar is 0.5 mm.

Tooth replacement never stops throughout the life of the animal (Figure 4B). Dental generations come one after another a number of times. This succession is achieved through the retention of a stem cell population in the dental lamina, allowing new teeth to develop at more or less regular intervals (Juuri et al., 2013).

##### Oligophyodonty

The frequency of tooth replacement undergoes drastic reduction relative to sister clades and replacement may stop after a few generations (Edmund, 1969). This condition has been described in fossil stem mammal clades (Luo et al., 2004) as well as in some agamid and tuatara species (Edmund, 1969; Cooper et al., 1970). Oligophyodonty seems associated with a delay in the initiation of the dental lamina during tooth replacement cycles (Handrigan et al., 2010) and remains of the dental lamina have been observed after the cessation of the replacement (Edmund, 1960). Nonetheless, most descriptions of oligophydonty do not present strong evidence of this cessation, suggesting that this term may not be relevant.

##### Diphyodonty

Dentition is limited to 2 dental generations over the life of the animal (Figure 4A). The interruption of tooth replacement is caused by a resorption of the dental lamina after the second generation has started its development (Dosedělová et al., 2015).

##### Monophyodonty

Primary dentition is never replaced over the life of the animal. The dental lamina may be maintained for some time in certain species, but it will not produce any subsequent teeth (Edmund, 1960; Buchtová et al., 2013; Dosedělová et al., 2015). The primary dentition may produce functional teeth or not. If the odontogenic germs of the first generation abort then monophyodonty will result in functional edentulism (see immediately below).

##### Anodonty and functional edentulism

It is important to distinguish vertebrates that completely lost the ability of developing teeth (anodonty) from those in which tooth development is still initiated until various odontogenetic stages, but without producing any functional teeth (functional edentulism). The first situation corresponds to the cases of modern birds (Louchart and Viriot, 2011), whereas the second situation corresponds with what has been reported for instance in the platypus or baleen whales (see section “Tooth Replacement”).

#### Path of the Replacement Tooth

The replacement path goes from where the replacement tooth develops to its final functional position. Concerning marginal dentitions, the replacement tooth always develops from the dental lamina that lies lingual to the base of the functional tooth.

##### Labio-vertical replacement

The replacement tooth develops in lingual position with respect to the base of the preceding tooth (Figures 4A,B,D). The newly formed tooth then migrates labialy to a position below the functional tooth while completing its development and mineralization. The eruption vector of the newly formed tooth is predominantly vertical. In Squamata, this dental replacement path was named the “iguanid method” by Edmund (1960).

##### Labio-mesial replacement

The replacement tooth develops and mineralizes in a disto-lingual position with respect to the preceding tooth. The eruption of the newly formed tooth occurs according to predominantly horizontal movements directed toward labio-mesial direction (Figures 4B,C). In Squamata, this dental replacement path was named “varanid method” by Edmund (1960).

Both the labial-vertical and labial-mesial eruptions correspond to extreme situations, and intermediates situation may exist (Cooper, 1966).

#### Resulting Resorption Patterns

While growing or erupting, the replacement tooth may drive resorption either on neighboring bone or on the preceding functional tooth.

##### Presence of resorption pits

The growth and eruption of the replacement tooth provokes a progressive resorption of the preceding tooth. A resorption pit develops on the functional tooth at the vicinity of the developing tooth. The pit can reach the pulp cavity, thus allowing the replacement tooth to settle in the pulp cavity of its predecessor during final developmental stages of the replacement tooth (Edmund, 1960). Histological studies of ankylosed teeth suggest that the alveolar bone is also resorbed through the replacement process (Caldwell et al., 2003; LeBlanc and Reisz, 2013; Haridy et al., 2017). By weakening the attachment of the preceding tooth, resorption may facilitate its shedding (Edmund, 1960). Labial-vertical tooth replacement always involves the development of resorption pits.

##### Absence of resorption pits

The growth and eruption of the replacement tooth do not provoke localized resorption of the preceding tooth. Once the replacement tooth is developed and mineralized, the preceding tooth is shed and the new tooth takes its place (Edmund, 1960).

#### Mode of Tooth Replacement

Amniote teeth are not replaced at random positions. Instead, teeth are replaced spatially and temporally through organized patterns (Edmund, 1960, 1969; Osborn, 1975). These patterns of tooth replacement can be classified in two main modes.

##### Sequential pattern

The sequential mode refers to a sequence that occurs at contiguous positions, which allows teeth to be replaced one after the other along the jaw. When considering a series of adjacent teeth, a gradient can be observed in the developmental stage of the various replacement teeth from one position to another, like in the springbok (*Antidorcas marsupialis*) (Smith, 2000). In the case of a mesio-distal gradient, anterior replacement teeth are more advanced than posterior teeth. In the case of a disto-mesial gradient, it is the opposite. This gradient defines a replacement wave.

##### Alternate pattern

The alternate mode refers to two waves of dental replacement that overlap along the jaw, the first impacting even positions and the second odd positions, as this was described in the green lizard (*Lacerta viridis*) (Berkovitz, 2000). These two waves are shifted in time. This results in a tooth replacement that never occurs at the same time for two adjacent positions (Edmund, 1960, 1969; Berkovitz, 2000). In the same way as for the sequential mode, this replacement mode may affect the functional dentition according to a mesio-distal or a disto-mesial direction.

## Diversity of Tooth Attachment, Implantation and Replacement in Extant Amniotes

Amniota can be divided in two main groups, namely the Synapsida and the Sauropsida. Currently living Synapsida are solely represented by Mammalia. Among Sauropsida, extant species of Aves and Testudines will not be mentioned here because they lost the ability to develop a dentition over the course of evolution (Louchart and Viriot, 2011; Schoch and Sues, 2016). Extant toothed sauropsids thus are the Crocodilia (crocodiles, alligators, and gharials) and Lepidosauria (tuataras, lizards, worm lizards, and snakes).

### Tooth Implantation and Attachment

#### The Mammalian Thecodont Gomphosis

Teeth of almost all mammals have thecodont implantation and gomphosis attachment (McIntosh et al., 2002). In species with brachyodont dentitions (e.g., *Homo*, *Sus*), dental roots are higher than crowns and only the radicular dentine is covered with cementum so that roots are attached to the bone through a ligament. In hypsodont mammals (e.g., *Equus*, *Loxodonta*), the crown continues to grow for some time before the roots develop and mark the end of tooth development. As a result, the part of the crown that stands in the alveolus is higher than the outside part. In hypselodont mammals (e.g., *Oryctolagus*, *Microtus*), the crown continues to grow in a very deep alveolus during the whole life of the animal and roots never develop. Roots are thus temporarily or definitively missing in hypsodont and hypselodont teeth. Despite dramatic changes in this crown-root arrangement, cementum is deposited either directly on crown enamel or on crown dentin when enamel is missing. Some studies were performed to evaluate differences in dental attachment between brachyodont, hypsodont, and hypselodont teeth. A comparison of dental attachment in hypsodont *versus* hypselodont molars in voles showed that the ligament attaching *Microtus* evergrowing molars does not show significant differences when compared to the ligament attaching roots of *Clethrionomys* hypsodont molars (Phillips and Oxberry, 1972). Differences in dental attachment between brachyodont and hypselodont teeth also were investigated in mouse by comparing the “rooted portion” of the evergrowing incisor with the anatomical roots of molars (Rooker et al., 2010). Results showed that brachyodont and hypselodont teeth were attached through histologically similar periodontal ligaments, but differences were observed in the distribution of Wnt responsive cells in the incisor periodontal ligament, which coincided with areas of periodontal ligament cell proliferation (Rooker et al., 2010). This suggests that continuous tooth growth has a great impact on tooth geometry and alveolus depth, but little impact on the nature of the attachment tissues. Gomphosis may, however, be impaired in mammals and these pathological ankyloses are particularly studied in human dentitions in which a gradual disappearance of the soft ligament leads to root ankylosis (Biederman, 1962; Andersson et al., 1984). The origin of root ankylosis is thought to arise from periodontal ligament injury (Biederman, 1962; Atrizadeh et al., 1971; Andersson et al., 1984; Rubin et al., 1984).

#### Mammalia and Crocodilia Display Many Similarities

Mammalia and Crocodilia display similar tooth implantations. Teeth are implanted through thecodonty into alveoli and their roots are higher than their crowns. The nature of their periodontium is also equivalent, involving a non-mineralized ligament mainly composed of collagen fibers. In both Crocodilia and Mammalia, the HERS is localized all around the root during its development in a comparable manner. The periodontium of Crocodilia, however, includes mineralized areas unlike the mammalian periodontium, as reported from a comparison between caiman and mouse (McIntosh et al., 2002). Another difference is that periodontal fibers are continuous through the bone of interdental septa in Mammalia (Cohn, 1972, 1975), whereas they penetrate superficially the bone of the interdental septa in Crocodilia (Berkovitz and Sloan, 1979). Thecodont implantation is, however, not the absolute rule in Crocodilia and Mammalia. For instance, teeth of porpoises (Phocoenidae) are implanted through aulacodont geometry (Figure 3B). Aulacodonty also occurs at the back of the jaws of juvenile *Caiman sclerops* and *Crocodylus niloticus* (Miller, 1968; Dumont et al., 2016). In *Caiman sclerops*, teeth that are located in the groove are attached by gomphosis, but both fiber organization and cementum structure differ from those observed for teeth attached in individual alveoli (Miller, 1968). Fibers that together form the ligament in the groove are denser and less organized than those observed in alveoli, especially between teeth and near the jaw bone where they form a dense interwoven fiber net. The cementum that covers teeth located into the groove also shows differences, as it is thicker on the mesial and distal faces of the roots.

#### Variation in Tooth Implantation and Attachment in Lepidosauria

**FIGURE 5:**
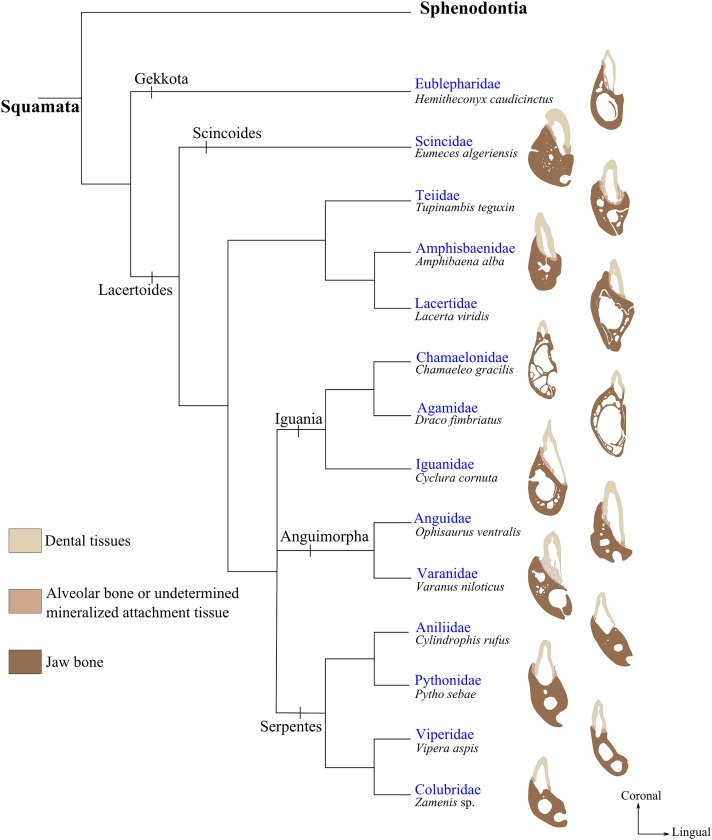
Implantation geometries placed next to the phylogenetic tree of squamates modified from (Pyron, 2016; Zheng and Wiens, 2016). Specimens illustrated from top to bottom are: *Hemitheconyx caudicinctus* (MNHN 1943-150); *Eumeces algeriensis* (MNHN 1886-343); *Tupinambis teguxin* (MNHN 1967-96); *Amphisbaena alba* (MNHN 1943-137); *Lacerta viridis* (MNHN 1887-813); *Chamaeleo gracilis* (MNHN 1942-114); *Draco fimbriatus* (MNHN 1887-880); *Cyclura cornuta* (MNHN 1919-45); *Ophisaurus ventralis* (MNHN 1943-143); *Varanus niloticus* (MNHN 1921-260); *Cylindrophis rufus* (MNHN 1869-779); *Python sebae* (MNHN 1953-155); *Vipera aspis* (MNHN 1869-855); *Zamenis* sp. (MNHN 1869-789).

The dentition of the only current representative of Rhynchocephalia, *Sphenodon punctatus*, displays an acrodont implantation (Kieser et al., 2009; Jenkins et al., 2017). Most members of Squamata have teeth with pleurodont implantation, apart from Agamidae and Chamaeleonidae that present, respectively, mixed acrodont and pleurodont tooth implantations and exclusively acrodont implantation in others (Zaher and Rieppel, 1999). In Agamidae for instance, the most mesial teeth are pleurodont whereas all remaining distal teeth are acrodont. Teiidae also display variability in implantation geometry, and their dentition have been considered alternatively as pleurodont (Romer, 1956; Presch, 1974; Berkovitz and Shellis, 2017), or subthecodont (Edmund, 1969; Berkovitz and Shellis, 2017). The way teeth are implanted in *Tupinambis teguixin* illustrates the difficulty of accurately determining the geometry of implantation in Teiidae (Figures 2C, 5). The labial sides of the teeth are fused to the jaw bone, but all tooth bases are also surrounded by mineralized attachment tissue and the teeth are set in a groove with high labial and low lingual walls. The attachment tissue forms mesial and distal walls separating teeth from each other. The part of the tooth embedded in the attachment tissue forms a root-like structure between one third and one half of the total length of the tooth. Thus, according to the above terminology, tooth implantation geometry in *Tupinambis* is subthecodont.

The geometry of tooth implantation in Serpentes also has been the subject of much debate, alternatively considered as acrodont (Romer, 1956), thecodont (Bell, 1997; Lee, 1997), or pleurodont (Zaher and Rieppel, 1999). The bases in snake teeth (Figure 5) are either set at the top of the tooth-bearing element (e.g., in Viperidae and Colubridae) or variously covered by attachment tissue (e.g., in Pythonidae and Aniliidae). When a tooth is shed, there remains at its base a bony circle that is composed of attachment tissues (LeBlanc et al., 2017b), which form ridges of hard tissue mesially, distally, and labially. However, teeth are not directly attached to these structures and the geometry of dental implantation in Serpentes is therefore neither thecodont nor subthecodont. Zaher and Rieppel (1999) considered the teeth of certain snakes as pleurodont, but the presence of bony three-sided walls that support the tooth bases makes it difficult to equate this implantation geometry with pleurodonty. Therefore, and since the geometry of dental implantation in snakes is relatively homogeneous but difficult to assign to an existing geometry, further histo-morphological investigations of the tooth-bone interface will be required to remove these current ambiguities.

Dental implantation is thus much more diversified in Lepidosauria than in Crocodilia and Mammalia, and this is also true regarding tooth attachment. Although ankylosis is the most common dental attachment in Lepidosauria (Edmund, 1969), teeth of certain snakes and pygopodid lizards are attached through a localized fibrous hinge, which could be considered as a special case of zone-restricted gomphosis. This specific attachment type has been described in the snakes *Dasypeltis*, *Elachistodon*, *Scaphiodontophis*, *Xenopeltis*, *Liphiodium*, *Sibynophis*, *Lycophiodon*, *Mehelya* as well as in the pygopodid lizard *Lialis* (Patchell and Shine, 1986a,b; Savitzky, 1981, 1983). According to these authors, possessing hinged teeth allowed better grasping the hard scales of their prey.

### Tooth Replacement

Most amniote clades exhibit polyphyodont dental replacement with an alternate mode of replacement (Edmund, 1960; Osborn, 1975). Crocodilia all are polyphyodont and their dental replacement begins as early as *in ovo* embryonic development (Westergaard and Ferguson, 1990). Up to three dental generations of the same family may coexist in one alveolus (Figure 4B): the currently functional tooth, its immediate successor that is usually placed just below (for lower teeth) or just above (for upper teeth), and a second succession tooth that is developing at the bottom of the alveolus, lingual to the functional tooth. The path of the replacement tooth first consists of a labial migration that is marked by the occurrence of a lingual resorption pit at the base of the functional tooth (Dumont et al., 2016). The functional tooth later on is shed and the replacement tooth erupts vertically. It was reported that certain dental positions of *Alligator mississippiensis* stop being replaced on the left and right side symmetrically during ontogeny (Bellairs and Miles, 1960; Auffenberg, 1988). Erickson (1996) refuted this latter idea, arguing that dental lamina injuries seemed to be a more plausible cause for explaining interruptions in the continuous dental replacement.

Most lepidosaurians exhibit polyphyodonty (Berkovitz and Shellis, 2017). Some tooth positions in agamid lizards and *Sphenodon* are, however, not replaced (Kieser et al., 2009), and the whole dentition of Chamaeleonidae is never replaced (Berkovitz and Shellis, 2017). Teeth of Chamaeleonidae as well as those that are not replaced in Agamidae and *Sphenodon* all are implanted by acrodonty (Kieser et al., 2009; Berkovitz and Shellis, 2017; Haridy et al., 2017). By contrast, teeth of Agamidae that have pleurodont implantation undergo replacement (Cooper et al., 1970). The case of *Sphenodon* is very peculiar as it is the only case of an extant lepidosaur that shows some replacement of acrodont teeth. Indeed, part of the anterior dentition of hatchlings is replaced by one larger tooth, that will in its turn be replaced (Harrison, 1901; Rieppel, 1992; Berkovitz and Shellis, 2017). Teeth implanted by acrodonty thus are systematically monophyodont in squamates, and the replacement is very limited in *Sphenodon* (Harrison, 1901; Rieppel, 1992; Jones et al., 2012; Jenkins et al., 2017). Cooper et al. (1970) also suggested that tooth replacement of pleurodont teeth in Agamidae was slow. Handrigan et al. (2010) even considered pleurodont teeth of the agamid *Agama barbatus* as oligophyodont. To date, it has not been formally demonstrated that replacement of agamid pleurodont teeth stops completely and the putative oligophyodonty of Agamidae therefore remains an open question.

**FIGURE 6:**
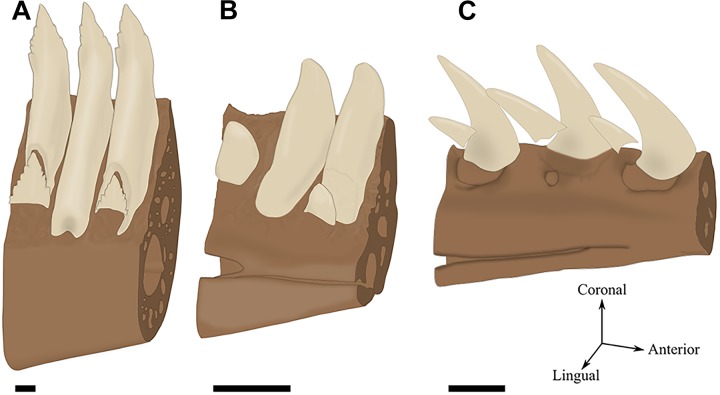
Path of the replacement tooth in Squamata. **(A)** Labio-vertical path in *Iguana iguana* (specimen MNHN 1939-523). **(B)** Intermediate path between **(A,C)** in *Ophiodes striatus* (specimen MNHN 1943-142). **(C)** Labio-mesial path in *Zamenis* sp. (specimen MNHN 1969-789). Scale bar is 0.4 mm.

Lepidosauria exhibit two main paths of dental replacement, namely the iguanid and varanid types (Edmund, 1960) (see section “Path of the Replacement Tooth”). The iguanid type is (Figure 6A) recognized in a large majority of species belonging to Iguania, Gekkota, Scincoidea, and Lacertoidea, whereas the varanid type (Figure 6C) is mainly found in Serpentes as well as in the anguimorphan families Varanidae, Helodermatidae, and Lanthanotidae (McDowell and Bogert, 1954; Edmund, 1960; Cooper, 1966; Rieppel, 1978). One peculiarity of the varanid path of dental replacement in Serpentes is that newly formed teeth are steeply inclined relative to functional teeth (Figure 6C) and crowns rotate toward the oral cavity as they erupt (Edmund, 1960; Rieppel, 1978). Mixed situations between the iguanid and varanid types may also exist. For example, in some teiids and two species of the genus *Amphisbaena*, an iguanid path of dental replacement is associated with a distal offset of replacement teeth, similar to what can be observed in the varanid type (Rieppel, 1978). Lastly, the dental replacement path is very variable in Anguidae (Figure 6B), in which iguanid, varanid, and mixed types have been reported (Edmund, 1960; Cooper, 1966; Rieppel, 1978). While dental replacement induces some resorption in Varanidae (Edmund, 1960; Rieppel, 1978), it involves little to no resorption in Helodermatidae and Lanthanotidae (Edmund, 1960).

Mammaliaformes is the only clade of amniotes that is characterized at its base by a transition from polyphyodonty to diphyodonty and from alternate to sequential tooth replacement (Luo et al., 2004). As a consequence, a vast majority of currently living mammals are fully or incompletely diphyodont. They develop a first generation that consists of a variable number of deciduous teeth, which are later replaced by a second generation composed of a variable number of permanent teeth. The permanent replacement tooth follows a labial-vertical path in most living mammals (Figure 4A), which causes substantial resorption of roots and crown bases of their deciduous predecessors. A trend toward reduction of dental replacement is clearly expressed within many mammalian orders, more frequently observed for certain dental loci than for a whole generation. For example, diphyodonty is highly repressed in Marsupialia as only one premolar locus is replaced while deciduous teeth are maintained in the other dental loci for the whole life of the animal (Luckett, 1993). Another prominent example of dental generation reduction is the monophyodonty of Muroidea, a tremendous rodent Superfamily that encompasses one fifth of the current mammalian diversity (Musser and Carleton, 2005). In Muroidea, only four deciduous incisors and twelve molars become functional during the whole life of the animal and no permanent tooth is ever developed (Gomes Rodrigues et al., 2011). However, extreme examples of functional edentulism reported for instance in Monotremata, Mysticeti, Manidae, and Myrmecophagidae do not correspond to more dramatic reduction (i.e., anodonty) because all these mammals incompletely develop at least a deciduous generation before being edentulous (Osborn, 1893; Green, 1937).

Luckett (1993) pointed out that the development of deciduous teeth, whether they abort or become functional, is a prerequisite for the development of teeth belonging to the permanent generation. This implies that molars likely are deciduous teeth whose development is more or less delayed and whose replacement was suppressed very early in the evolutionary history of mammals (Wilson, 1956; Luckett, 1993). However, 5 out of the *circa* 5500 extant species of Mammalia do replace their molars, which make these exceptions particularly rare. Indeed, *Petrogale concinna* (Diprotodontia), *Heliophobius argenteocinnereus* (Rodentia) and 3 species of *Trichechus* (Sirenia) independently acquired the ability of continuously replacing the most distal molar position in each dental row (Gomes Rodrigues et al., 2011). When a newly formed molar erupts vertically at the back of the row, it later on pushes forward all the teeth previously in place, thus causing a treadmill movement leading to inter-dental resorption as well as the shedding of the oldest tooth located at the front of the dental row. This mechanism allows a continuous dental replacement associated with horizontal (in *Petrogale* and *Trichechus*) to diagonal (in *Heliophobius*) paths of dental replacement, but it differs in its organization from the polyphyodonty described in other Amniota.

## Evolution of Tooth Attachment, Implantation and Replacement in Amniota

### Assessment of the Ancestral State

**FIGURE 7:**
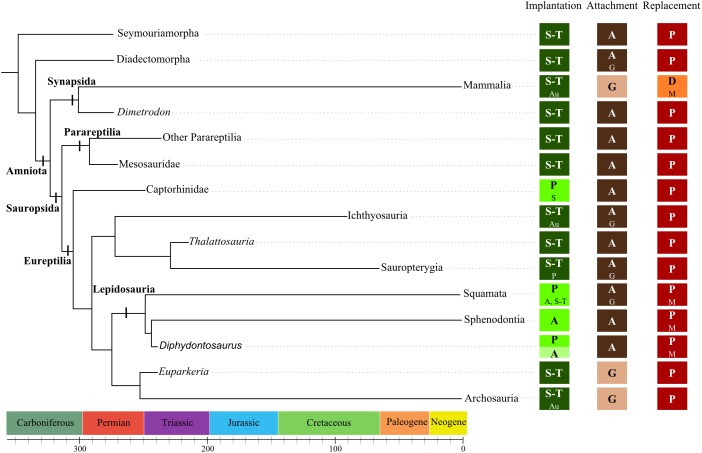
Dental features among early amniotes. Cladogram presenting some of the groups of stem or early Amniota. The dental features associated with each group are the assumed ancestral state for the groups or the described states for the genera (in bold). Other states that have been described in the groups are indicated with a smaller font. Implantation = geometry of implantation (S, subthecodonty; T, thecodonty; P, pleurodonty; A, acrodonty; Au, aulacodonty) Attachment = nature of the attachment (A, ankylosis; G, gomphosis) Replacement = number of replacement generation (P, polyphyodont; D, diphyodont; M, monophyodont). From (Romer and Price, 1940; Edmund, 1960, 1969; Ewer, 1965; Dong, 1972; Evans, 1984; Whiteside, 1986; Laurin and Reisz, 1995; Laurin, 1996b; Small, 1997; Rieppel, 2001; Cabreira and Cisneros, 2009; Dyke and Kaiser, 2011; Macdougall and Modesto, 2011; Pretto et al., 2012; LeBlanc and Reisz, 2013, 2015; Benton et al., 2015; Sassoon et al., 2015; de Miguel Chaves et al., 2018).

The first step in understanding the evolution of tooth implantation, attachment, and replacement in amniotes is to determine the ancestral state for these characters. This work required collecting data on the earliest fossil amniotes and/or in basal fossils belonging to their closely related groups. For Edmund (1960, 1969), the earliest amniotes were polyphyodont, they possessed protothecodont tooth implantation and the teeth were attached by ankylosis. Diadectomorphs and other stem groups like seymouriamorphs are the closest approximations for the hypothetical ancestral amniote (Laurin and Reisz, 1995; Ruta et al., 2003). Herbivorous diadectomorphs, such as *Diadectes* and its close relatives, had teeth with thecodont implantation and ankylosed attachment (LeBlanc and Reisz, 2013). Their periodontium included layers of cementum as well as alveolar bone, in which Sharpey fibers were visible. There was, however, no periodontal spaces, which suggested that no soft ligament was present in mature teeth. Seymouriamorphs such as *Ariekanerpeton sigalovi* had teeth implanted into alveoli, but attachment was not precisely described (Laurin, 1996a). It is worth noting that for Edmund (1969), protothecodont implantation in early amniotes required that the bases of each tooth were ankylosed into a more or less deep socket by the deposition of cement, which corresponds in our redefined terminology to either subthecodont or thecodont implantation, because the depth of the alveolus was not specified. We cannot consequently distinguish between subthecodonty and thecodonty in this case, because it is difficult to distinguish these two implantation geometries in fossil specimens without histological or microtomographic data. Finally, most basal groups of amniotes and their closely related groups possessed teeth implanted in shallow or deep alveoli (respectively, subthecodont or thecodont) and these teeth were attached through ankylosis (Figure 7). Dentitions of the earliest amniotes were continuously replaced in a labial-vertical path of dental eruption (Edmund, 1960).

### Evolution of Periodontal Tissues

**FIGURE 8:**
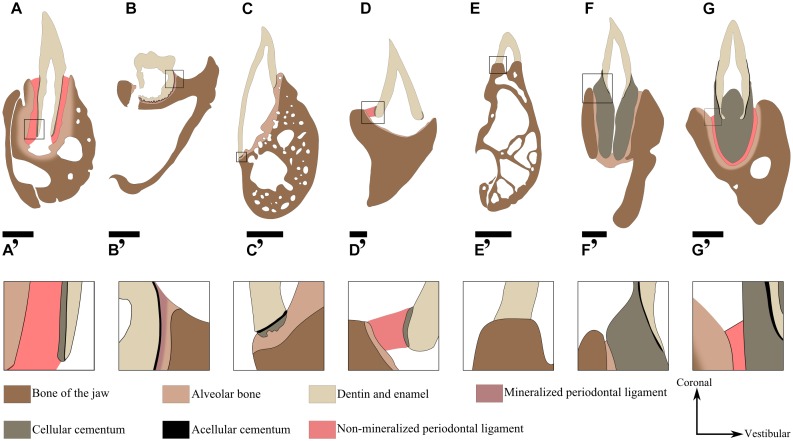
Current knowledge of the nature of the attachment tissues nature in different extinct and extant species represented from available literature. **(A)** *Crocodylus niloticus* (Archosauria, Crocodilidae) attachment is gomphosis. Scale bar is 2 mm. **(B)** *Diadectes* (Diadectomorpha) (LeBlanc and Reisz, 2013). Attachment is ankylosis. Scale bar is 1 cm **(C)** *Iguana iguana* (Squamata, Iguanidae) (Luan et al., 2009). Attachment is ankylosis. Scale bar is 1.5 mm **(D)** *Dinilysia* (Squamata, Dinilysiidae) (Budney et al., 2006). Attachment is hinged through soft ligament. Scale bar is 1 mm. **(E)** *Chamaeleo calyptratus* (Squamata, Chamaeleonidae) (Buchtová et al., 2013). The attachment is ankylosis, Scale bar is 0.8 mm. **(F)** *Platecarpus* (Squamata, Mosasauridea) (Caldwell, 2007). Attachment is ankylosis. Scale bar is 2 cm **(G)** *Platypterygius*, (Ichthyosauria) (Maxwell et al., 2011b). Attachment is gomphosis. The cellular cementum is vascularized (osteocementum). The panels **(A’–G’)** correspond to a magnification of the area framed on the panels **(A–G)**, respectively. Scale bar is 1 cm.

Although ankylosis is considered as the basal state for dental attachment in amniotes (Figure 7), the issue of the nature and arrangement of periodontal tissues is central to understand the evolution of tooth attachment as a whole. An important issue is the question of the homology between the “attachment bone” of non-crocodilian and non-mammalian amniotes and the periodontal tissues of crocodilians and mammals. The historical view (Peyer, 1968; Zaher and Rieppel, 1999; Gaengler, 2000) on this subject is that only Mammalia and Crocodilia have a complex tripartite periodontium that involves a cementum layer, a soft periodontal ligament, and a layer of alveolar bone (Figures 8A,A’). In contrast, teeth of early Amniota and Lepidosauria are considered to be directly ankylosed to the “attachment bone,” which is in turn attached to the bone of the jaw (Edmund, 1969; Zaher and Rieppel, 1999). However, periodontal tissues were recently described in fossil archosaurs, including theropod dinosaurs (Fong et al., 2016; LeBlanc et al., 2017a), toothed birds (Dumont et al., 2016), titanosaurid sauropods (García and Cerda, 2010; García and Zurriaguz, 2016), hadrosaurids (LeBlanc et al., 2016b, 2017a), and ceratopsids (LeBlanc et al., 2017a). These studies showed that the tripartite periodontium was present in all of these groups and presumably in the common ancestor of all dinosaurs and crocodilians as well. It was also present in various early synapsids such as *Dimetrodon*, dinocephalians, therocephalians (LeBlanc et al., 2016a) and in other diapsid reptiles, including ichthyosaurs (Maxwell et al., 2011b; Scheyer and Moser, 2011). As previously mentioned, even species of the stem amniote group Diadectomorpha possessed a tripartite periodontium (Figures 8B,B’) (LeBlanc and Reisz, 2013). Alveolar bone (but originally described as “attachment bone”) as well as cementum were also described in mesosaurs (Pretto et al., 2012), extinct and extant Squamata (Budney et al., 2006; Caldwell, 2007; Luan et al., 2009; LeBlanc et al., 2017b), and Rhynchocephalia (Kieser et al., 2009). This set of observations supports an alternative hypothesis: alveolar bone, cementum and the periodontal ligament were already present in early Amniota (LeBlanc and Reisz, 2013).

The confusion and debate over the relationship of “attachment bone” to the three attachment tissues that characterize the mammalian periodontium centers around a single question: what do we call the mineralized attachment tissues when a tooth is ankylosed to the jaws? Poole (1967) and Edmund (1969) assumed that cementum was homologous with lepidosaurian attachment bone based on observations made on snakes and lizard species. Poole noted that the acellular nature of the attachment bone resembles acellular cementum. The same assumption was made in mosasaurs and varanid lizards (Rieppel and Kearney, 2005; Kearney and Rieppel, 2006). LeBlanc and Reisz (2013) speculated that attachment bone was homologous with alveolar bone in diadectomorphs, thus joining Maxwell et al. (2011a) who also described attachment bone as alveolar bone in two species of the genus *Varanus*. Here we suspect that the tissue called “attachment bone” actually corresponds to different types of tissues depending on the studied groups. Further investigations of histological features of the attachment bone in a large collection of taxa are still necessary to assess the homologies of periodontal tissues among amniotes.

Among living squamates, various families of snakes and limbless lizards have dentitions including hinged teeth that are attached by a soft ligament (Savitzky, 1981, 1983; Patchell and Shine, 1986a,b; Maher and Kearney, 2006). The occurrence of hinged teeth was suspected in the fossil snake *Dinilysia* (Budney et al., 2006) based on morphological similarities with extant species of snakes possessing hinged teeth (Figures 8D,D’), and the fact that the teeth are almost always lost post-mortem, which strongly suggested that the teeth were held in place by soft tissues. However, no histological evidence of the former presence of a ligament could be gathered from the polished thick sections of the fossils.

As ligaments do not fossilize, the former presence of a soft periodontal ligament can be detected in thin section by checking for the existence of a periodontal space in the vicinity of the roots or by looking for ligament insertion traces (Sharpey’s fibers) in the cementum or alveolar bone (LeBlanc and Reisz, 2013). This type of detailed histological investigation demonstrating the former presence of a periodontal ligament has been documented for several ichthyosaurs (Maxwell et al., 2011b; Scheyer and Moser, 2011), mesosaurs (Pretto et al., 2012), pliosaurs (Sassoon et al., 2015), and mosasaurs (Caldwell, 2007; Luan et al., 2009; LeBlanc et al., 2017b). Shell-crushing Mosasauridae were notably shown to have a dental attachment comparable to that of crocodilians (LeBlanc et al., 2017b). The emergence of hinged teeth attached by a soft ligament has thus been achieved several times independently during the evolution of Squamata and this feature is clearly linked with the acquisition of a durophagous diet, including scincivory (Patchell and Shine, 1986b).

Further confounding the categorization of tooth attachment in amniotes is the recent finding that the presence of a soft periodontal ligament around the tooth does not necessarily mean that the teeth are permanently attached by gomphosis. On the contrary, it has recently been shown in some fossil amniotes that the ligament may remain unmineralized while the tooth was erupting and became functional, before gradually mineralizing until the tooth was completely ankylosed (LeBlanc and Reisz, 2013; LeBlanc et al., 2016a). A progressive mineralization of the periodontal ligament resulting in a complete ankylosis of the tooth was described in diadectomorphs (Figures 8B,B’) (LeBlanc and Reisz, 2013), mosasaurs (Figures 8F,F’) (Luan et al., 2009; LeBlanc et al., 2017b), and in three extinct relatives of mammals (LeBlanc et al., 2016a). Ankylosis in this context is therefore intimately related to gomphosis in histological terms. One might even wonder whether mammalian or archosaurian gomphosis with a soft ligament could be derived from a delay or absence of ligament mineralization, thus highlighting a paedomorphic trend in the evolution of dental attachment. Indeed, one line of evidence in support of this notion is the lack of mineralization of the periodontal ligament as investigated in the mouse (*Mus musculus*) and the caiman (*Caiman crocodilus*), which was putatively related to the retention of epithelial rests of Mallassez (McIntosh et al., 2002; Luan et al., 2006, 2009; LeBlanc et al., 2016a).

Tooth ankylosis in lepidosaurians can also differ from the ankylosis of the ligament by mineralization discussed above. A second type of ankylosis includes substantial variations in the structure of the periodontium. Buchtová et al. (2013) showed that tooth attachment in the veiled chameleon (*Chamaeleo calyptratus*) was achieved by the fusion of dental predentin with the tooth-bearing element (Figures 8E,E’), and that neither cementum nor ligament could be observed. Other studies of the dentition in acrodont (*Sphenodon punctatus*) and pleurodont (*Iguana iguana*, *Varanus niloticus*) lepidosaurians revealed that cementum was present as acellular layers in contact with a mineralized attachment tissue (Kieser et al., 2009; Luan et al., 2009; Maxwell et al., 2011a) (Figures 8C,C’), called alveolar bone in the case of *Varanus niloticus* (Maxwell et al., 2011a). Despite teeth of *Chameleo* and *Sphenodon* both have acrodont implantations, attachment displays various histological characteristics in these two genera.

Other studies have highlighted the variations observed in the organization of the tripartite periodontium. Diverse studies have investigated the periodontal histology in mosasaurs (Caldwell et al., 2003; Luan et al., 2009). Five different periodontal tissues were identified notably in mosasaurs; namely a layer of acellular cementum, a layer of vascularized cellular cementum (referred as osteocementum), a mineralized periodontal ligament, an interdental ridge (composed of alveolar bone), and bone belonging to the tooth-bearing element (Luan et al., 2009). All these tissues were also described in *Platecarpus*, apart from the mineralized ligament (Figures 8F,F’) (Caldwell et al., 2003). When compared to what is known from the periodontium of mammals or crocodilians, the main differences lie in the presence of both a thick osteocementum cone that provides most of the dental anchorage, and a mineralized ligament. The periodontium of mosasaurs is comparable to that of Diadectomorpha, except for the presence of the thick layer of osteocementum. Osteocementum was also demonstrated in the periodontium of aulacodont ichthyosaurians (Maxwell et al., 2011b), such as *Platypterygius* (Figures 8G,G’). The term ankylosis is thus currently used to describe many types of fused dental attachment, involving different types of mineralized tissue at the interface between the tooth and the tooth-bearing element. Additional studies of attachment through ankylosis will be necessary to better understand the evolutionary dynamics of tooth attachment in amniotes. These studies will have to consider not only the attachment of the functional tooth (i.e., gomphosis versus ankylosis), but also the developmental origin of this attachment tissue as well as the arrangement of the different tissues that compose the periodontal complex.

### Evolution of the Implantation Geometry

As discussed in Section “Assessment of the Ancestral State,” teeth of most basal amniotes were implanted in shallow or deep sockets, so that the plesiomorphic state for dental implantation in amniotes was probably subthecodonty or thecodonty. Again, it is important to note here that this refers only to the geometry of the attachment and not the identity of the tissues that formed the attachment, which were probably cementum, alveolar bone, and the periodontal ligament (mineralized or unmineralized). From this state on, new dental implantations appeared over the course of evolution among various taxa.

Pleurodont implantation, which consists of a drastic reduction of the lingual wall of the tooth-bearing element, likely was the basal condition of tooth implantation in Lepidosauria and most of the current species of lizards still have pleurodont teeth (Edmund, 1969). Yet, a secondary trend toward a partial or complete acquisition of acrodont implantation emerged over the evolution of rhynchocephalians (Jenkins et al., 2017). While the earliest known rhynchocephalian *Gephyrosaurus* (Evans, 1980, 1981) had teeth exclusively implanted through pleurodonty, teeth of the more derived *Diphydontosaurus* displayed pleurodont implantations in the mesial half of the jaw and acrodont implantations in the distal half (Whiteside, 1986). Teeth of certain squamates, such as acrodontan iguanians also displayed acrodont implantation, which probably stemmed from a pleurodont ancestor (Zaher and Rieppel, 1999; Smirina and Ananjeva, 2007). In acrodontan iguanians (Chamaeleonidae and Agamidae), the earliest fossils had pleurodont teeth mesially while their distal teeth were implanted through an intermediary stage between pleurodonty and acrodonty (Simões et al., 2015). These data confirm that pleurodonty likely was the plesiomorphic condition among Lepidosauria (Evans, 1984; Jenkins et al., 2017), and that acrodonty emerged independently several times in various lineages (Whiteside, 1986; Simões et al., 2015; Jenkins et al., 2017). This phenomenon has also been observed in other extinct reptilian lineages. Acrodonty was described in the captorhinid *Opisthodontosaurus* (Reisz et al., 2015; Haridy et al., 2017) while the most basal captorhinids had pleurodont tooth implantation (LeBlanc and Reisz, 2015).

Thecodonty is the predominant form of tooth implantation in all mammals and archosaurs (Edmund, 1969; Zaher and Rieppel, 1999). Thecodonty also was the prevailing tooth implantation geometry in the reptilian lineage Sauropterygia (Rieppel, 2001). Some members of the Thalattosauria, a close sister group of Sauropterygia, exhibited a subthecodont implantation associated with ankylosed attachment (Rieppel, 2001). As subthecodonty (or thecodonty) is the assumed implantation geometry for basal amniotes, tooth implantation in Sauropterygia and Ichthyopterygia probably represents a symplesiomorphy. Thecodonty in Sauropterygia was predominantly associated with ankylosed attachment, with the exception of procumbent mesial teeth of *Placodus* that were attached by gomphosis into deep alveoli (Jaekel, 1907; Rieppel, 2001) as well as teeth of pliosaurs that also were attached by gomphosis (Sassoon et al., 2015). Only one species of filter-feeding Eosauropterygia was recently shown to have teeth implanted by pleurodonty (de Miguel Chaves et al., 2018).

Tooth implantation geometry in Mosasauridae has been thoroughly debated between supporters of a pleurodont implantation (Zaher and Rieppel, 1999) and those of a thecodont implantation (Caldwell, 2007; LeBlanc et al., 2017b). While Zaher and Rieppel (1999) considered that mosasaurids displayed a derived type of pleurodonty, Caldwell (2007) and Luan et al. (2009) demonstrated that teeth were implanted into deep alveoli, and that dental roots were mainly composed of vascular cementum. Associated attachment was mainly provided by ankylosis of a mineralized periodontal ligament (Caldwell et al., 2003; Luan et al., 2009; LeBlanc et al., 2017b), but gomphosis was also described in some mosasaurids (Polcyn et al., 2010; LeBlanc et al., 2017b). Finally, the thecodont dental implantation of mosasaurids likely was derived from the plesiomorphic state of pleurodont implantation in other squamates. Thecodont tooth implantation was also reported as the prevalent mode of dental implantation in Ichthyopterygia during the Middle Triassic (Motani, 1997). Some of the oldest ichthyopterygian fossils such as *Utatsusaurus hataii* had dentitions implanted through subthecodonty associated with ankylosed attachment (Motani, 1997). Motani (1997) even named “ichtyosaurian thecodonty” an original implantation type that he found only in two genera of ichthyosaurs (*Cymbospondylus*, *Shonisaurus*), and which consisted of an ankylosis of the deepest part of the roots. Aulacodonty arose later on during the evolution of Ichthyosauria and this dental implantation became dominant in Upper Triassic and post-Triassic genera (Motani, 1997). Aulacodonty also emerged independently in archosaurs and mammals. Within archosaurs, the fossil bird *Hesperornis regalis* (Dumont et al., 2016) had teeth permanently implanted through aulacodonty whereas only the dentition of juveniles in *Caiman crocodilus* and the extinct toothed bird *Ichthyornis dispar* display transitory aulacodont implantation before transitioning to thecodonty in adults (Miller, 1968; Dumont et al., 2016). Within mammals, aulacodont implantation was discovered in the cetacean *Delphinus delphis* and is thought to have originated from thecodonty (Mazin, 1983).

### Evolution of Tooth Replacement

Polyphyodonty is undoubtedly the basal mode of dental replacement in Amniota and this pattern persisted in most sauropsids and non-mammalian synapsids (Berkovitz, 2000; Pough et al., 2009). Many exceptions are, however, reported, notably among squamates and rhynchocephalians. As already discussed concerning tooth implantation in fossil rhynchocephalians (see section “Evolution of the Implantation Geometry”), *Gephyrosaurus* had exclusively pleurodont teeth (Evans, 1980) whereas *Diphydontosaurus* had pleurodont mesial teeth and acrodont distal teeth (Whiteside, 1986). Dentitions of *Gephyrosaurus* underwent continuous replacement and only pleurodont teeth of *Diphydontosaurus* were continuously replaced whereas acrodont distal teeth remained monophyodont. In an identical manner, teeth implanted through acrodonty in chamaeleonid and agamid squamates are never replaced, whereas the pleurodont teeth are continually replaced (Cooper et al., 1970). Put together, these data led to the hypothesis that teeth implanted through acrodonty were never replaced throughout life (Zaher and Rieppel, 1999; Smirina and Ananjeva, 2007). Some authors even suggested that the absence of dental replacement should be a way to recognize acrodont teeth (Zaher and Rieppel, 1999). These observations as well as the resulting hypothesis are, however, conceivable only for squamates, because the acrodont teeth of the captorhinid *Opisthodontosaurus* exhibited polyphyodonty (Reisz et al., 2015; Haridy et al., 2017). Acrodont implantation is thus not universally associated with monophyodonty among amniotes. We recommend that additional comparative studies be conducted to understand why acrodont teeth are generally not replaced in lepidosaurians while they are replaced in these captorhinids.

Teeth of acrodont lepidosaurians show remarkable adaptations to resisting abrasion. Studies on the dentition of *Uromastyx aegyptia* revealed that tooth pulp chambers were progressively filled with secondary dentin and that the bone supporting tooth bases became accordingly more compact during ontogeny (Throckmorton, 1979). Another study on the dentition of *Chamaeleo calyptratus* demonstrated that acrodont teeth became firmly attached to the jaw bone by mineralized attachment tissue and that pulp cavities were progressively filled with dentin over life, so that teeth finally merged and together formed a single functional unit (Dosedělová et al., 2016). These original adaptations, which constitute various ways of preventing tooth breakage or tooth loss over life, are probably related to prolonging the life of a monophyodont dentition (Throckmorton, 1979).

Even though most acrodont lepidosaurians, such as chamaeleonids, do not replace their teeth, newly formed teeth still develop at the distal end of the dental rows as tooth-bearing elements continue to grow throughout ontogeny (Cooper et al., 1970; Kieser et al., 2009 ; Dosedělová et al., 2016). Cooper et al. (1970) suggested that the addition of larger teeth at the rear of the dental rows in monophyodont lizards would be a way to maintain appropriate sized teeth in continuously growing jaws. In certain polyphyodont groups like crocodilians, teeth became gradually larger as successive replacements are made, which allow the dentition to remain appropriately sized for the growing skull throughout ontogeny (Brown et al., 2015). In other groups, like non-varanid squamates such as iguanas (Edmund, 1969), the number of teeth at the rear of dental rows increased while skull growth (Brown et al., 2015). There are therefore two ways of maintaining functional correspondence between teeth and growth of tooth-bearing elements: either replacement teeth become larger and larger, or new dental positions appear at the rear of dental rows, this latter being the pattern observed in some acrodont lepidosaurians.

Evolution of dental replacement in Synapsida makes up another substantial exception to the widespread polyphyodonty in amniotes. Even if basal synapsids still replaced their teeth continuously, a transition from polyphyodonty to diphyodonty occurred about 200 million years ago and is documented by a rich fossil record (Luo et al., 2004). Among non-mammalian synapsids, continuous dental replacement was reported in *Dimetrodon* (Romer and Price, 1940), dicynodonts (Hopson, 1964), therocephalians (Kermack, 1956; Hopson, 1964), gorgonopsians (Kermack, 1956), and non-mammalian cynodonts (Hopson, 1964; Luo et al., 2004). *Thrinaxodon* is often taken as an example to illustrate the pre-mammalian situation, in which all tooth positions are continuously replaced according to an alternate pattern (Luo et al., 2004). According to Kermack, the alternate, continuous replacement transitioned to sequential replacement in Therocephalia and Gorgonopsia, but no formal evidence has been produced to date. The basal mammaliaform *Sinoconodon* had diphyodont replacement for premolars and molars, but at least three dental generations for canines, which constituted a reduced tooth replacement rate compared to basal synapsids (Luo et al., 2004). These data indicate (1) that the transition from polyphyodonty to diphyodonty did not occur through a coordinated decrease in the replacement rate of all dental positions within the dentition, but rather that (2) the decrease in the replacement rate firstly took place in postcanine positions. Diphyodonty as it is known in currently living mammals could have occurred for the first time in the mammaliaform *Morganucodon* (Luo et al., 2004). Thus, the synapsid fossil record shows a progressive evolutionary reduction of the total number of dental generations across these major clades. This reduction to a limited number of dental generations can be associated with the evolution of precise occlusion related to the increase in complexity of the occlusal surfaces of teeth in mammals (Jernvall and Thesleff, 2012).

Some cynodonts belonging to the family Tritylodontidae displayed an altered polyphyodonty. Their postcanine teeth were not replaced according to the labial-vertical path, as is the case in most synapsids, but newly formed teeth were regularly added at the rear of each dental row (Crompton, 1972; Sues, 1985; Luo et al., 2004). It has even been shown that the newly formed teeth exhibited post-eruptive mesial horizontal movements in *Bienotherium* (Matsuoka and Setoguchi, 2000) and *Tritylodon* (Jasinoski and Chinsamy, 2012). This type of continuous tooth replacement, which involved a treadmill-like system, strongly resembled that described in the modern mammals *Heliophobius*, *Petrogale*, and *Trichechus* (see section “Tooth Replacement”). In contrast to the polyphyodonty observed in other amniotes, this treadmill-like replacement system allowed maintaining a relatively precise occlusion between teeth despite changes in the respective positions of teeth throughout the life of the animal. This type of replacement was considered to be associated with a diet consisting of highly abrasive intakes (Gomes Rodrigues et al., 2011) and/or herbivorous diet (Kühne, 1956; Hu et al., 2009).

## Conclusion

This article reviews much of the historical and modern work on attachment, implantation and replacement of teeth in amniotes. We propose here synthetic definitions or redefinitions of the most commonly used terms, while seeking to remove ambiguities about terms that could be confusing. Similarly, we have also tried to clarify why some ideas that have settled over time are clearly no longer relevant today. As an example, recent research demonstrates that there is no concrete link the absence of tooth replacement and the acrodont dental implantation.

The three classical types of dental implantation (i.e., thecodonty, acrodonty, and pleurodonty) are obviously not sufficient to describe the diversity of tooth implantation types seen in extant and extinct species of amniotes, and the terminology we propose here seems to minimally address this diversity. However, as we show here, even the traditional usage of these three terms has become conflated with other aspects of the development and evolution of teeth. Thecodonty was long considered as the typical and exclusive dental implantation for both Archosauria and Synapsida (Edmund, 1969; Zaher and Rieppel, 1999; LeBlanc et al., 2017a) and various authors considered thecodonty as universally associated with a gomphosis (Edmund, 1969; Zaher and Rieppel, 1999), or even synonymous with the presence of tripartite periodontium, especially alveolar bone (Caldwell et al., 2003; Budney et al., 2006; Pretto et al., 2012; LeBlanc and Reisz, 2013). In the Section “Evolution of Periodontal Tissues,” we pointed out that a tripartite periodontium appeared very early during the evolution of amniotes, and that it was not associated with gomphosis. Thecodonty is also not only characteristic of the archosaurs and mammals, but has instead appeared many times independently in various other groups, including Ichthyopterygia, Sauropterygia, and Mosasauridae. In general, dental implantation shows significant variations during the evolution of amniotes, which limits the phylogenetic signal conveyed by this character.

These findings contrast strongly with the evolutionary history of tooth attachment modes in amniotes and the histological features of dental attachment are promising sources for phylogenetically informative characters. Recent studies on tooth attachment have demonstrated that amniotes show a remarkably consistent set of periodontal tissues, even among their most basal members, but that the amounts, arrangements, and degrees of mineralization of these tissues vary dramatically. Historically, it was thought that crocodilians and mammals independently evolved a tripartite periodontium and the dental gomphosis, but the presence of similar tissues in the earliest stem and crown amniotes is now reframing the origins of the amniote periodontium. Based on recent literature, we can hypothesize that the three periodontal tissues that characterize dental attachment in amniotes already were present in the earliest representatives of the group and the first occurrence of these tissues is to be found among the evolutionary history of non-amniote vertebrates. This places some peculiar observations of tooth attachment tissues in some non-amniote vertebrates into clearer context. For example, teeth of some extant actinopterygians are reportedly composed of a shallow alveolar socket, a periodontal ligament and acellular cementum (Soule, 1969). In the same vein, tooth attachment in most lissamphibian involves the presence of a non-mineralized ligament between teeth and their bases (Davit-Béal et al., 2007). However, the precise nature of attachment in most non-amniote vertebrates is mostly unknown, partly due to the widespread use of the term “bone of attachment,” and this would require deeper investigation to understand the evolution of the periodontium outside of amniotes.

From the amniote basal state of dental attachment through ankylosis, different evolutionary pathways can be observed. Loss or modifications of certain periodontal tissues led to the appearance of derived types of ankylosis (Buchtová et al., 2013). The retention of a non-mineralized periodontal ligament allowed for the development of gomphosis (LeBlanc et al., 2016a). We suggest that the study of tooth attachment be conducted through a clear identification of periodontal tissues via histological sectioning. This represents a challenge concerning studies of fossil specimens, because fossils are sometimes rare, which limits the possibilities to perform invasive techniques. Non-destructive techniques such as X-ray microtomography can be a valuable tool in this context. This will provide more relevant information to help identify possible homologies compared to studying external anatomy and tooth implantation geometry alone (LeBlanc et al., 2017a).

## Author Contributions

This study was initiated by BT-P and LV. Preliminary observations using X-ray microtomography were made by TB, BT-P, and LV. Data collection from literature were made by TB, BT-P, LV, and AL. TB, BT-P, LV, AL, and MC contributed in writing the main text. All authors contributed to manuscript revision, read and approved the submitted version.

## Conflict of Interest Statement

The authors declare that the research was conducted in the absence of any commercial or financial relationships that could be construed as a potential conflict of interest.
